# Pan-inhibition of the three H_2_S synthesizing enzymes restrains tumor progression and immunosuppression in breast cancer

**DOI:** 10.1186/s12935-024-03317-1

**Published:** 2024-04-16

**Authors:** Alyaa Dawoud, Rana A. Youness, Heba Nafea, Tamer Manie, Carole Bourquin, Csaba Szabo, Reham M. Abdel-Kader, Mohamed Z. Gad

**Affiliations:** 1 Biochemistry Department, Faculty of Pharmacy and Biotechnology, German University in Cairo, Cairo, Egypt; 2 Molecular Genetics and Biochemistry Department, Faculty of Biotechnology, German International University (GIU), New Administrative Capital, Cairo, Egypt; 3 Department of Breast Surgery, National Cancer Institute, Cairo University, Cairo, Egypt; 4 School of Pharmaceutical Sciences, Institute of Pharmaceutical Sciences of Western Switzerland, Department of Anaesthesiology, Pharmacology, Intensive Care and Emergency Medicine, University of Geneva, Geneva, 1211 Switzerland; 5 Chair of Pharmacology, Section of Science and Medicine, University of Fribourg, Fribourg, 1700 Switzerland; 6 Pharmacology and Toxicology Department, Faculty of Pharmacy and Biotechnology, German University in Cairo, Cairo, Egypt

## Abstract

**Background:**

Hydrogen sulfide (H_2_S) is a significant endogenous mediator that has been implicated in the progression of various forms of cancer including breast cancer (BC). Cystathionine-β-synthase (CBS), cystathionine-γ-lyase (CSE), and 3-mercaptopyruvate sulfurtransferase (3MST) are the three principal mammalian enzymes responsible for H_2_S production. Overexpression of CBS, CSE and 3MST was found to be associated with poor prognosis of BC patients. Moreover, H_2_S was linked to an immune-suppressive tumor microenvironment in BC. Recently it was observed that BC cells, in response to single or dual inhibition of H_2_S synthesizing enzymes, develop an escape mechanism by overexpressing alternative sources of H_2_S generation. Thus, the aim of this work is to escape the H_2_S compensatory mechanism by pan repressing the three enzymes using microRNAs (miRNAs) and to investigate their impact on the oncogenic and immunogenic profile of BC cells.

**Methods:**

BC female patients (*n* = 25) were recruited. *In-silico* analysis was used to identify miRNAs targeting CBS, CSE, and 3MST. MDA-MB-231 cells were cultured and transfected using oligonucleotides. Total RNA was extracted using Biazol, reverse transcribed and quantified using qRT-PCR. H_2_S levels were measured using AzMc assay. BC hallmarks were assessed using trans-well migration, wound healing, MTT, and colony forming assays.

**Results:**

miR-193a and miR-548c were validated by eight different bioinformatics software to simultaneously target CBS, CSE and 3MST. MiR-193a and miR-548c were significantly downregulated in BC tissues compared to their non-cancerous counterparts. Ectopic expression of miR-193a and miR-548c in MDA-MB-231 TNBC cells resulted in a marked repression of CBS, CSE, and 3MST transcript and protein levels, a significant decrease in H_2_S levels, reduction in cellular viability, inhibition of migration and colony forming ability, repression of immune-suppressor proteins GAL3 GAL9, and CD155 and upregulation of the immunostimulatory MICA and MICB proteins.

**Conclusion:**

This study sheds the light onto miR-193a and miR-548c as potential pan-repressors of the H_2_S synthesizing enzymes. and identifies them as novel tumor suppressor and immunomodulatory miRNAs in TNBC.

**Supplementary Information:**

The online version contains supplementary material available at 10.1186/s12935-024-03317-1.

## Introduction

H_2_S plays a complex role in cancer. It has both pro-tumor and anti-tumor effects depending on the concentration of H_2_S, the source of H_2_S (exogenous vs. endogenous) and the cancer model used [1–4]. H_2_S – especially at lower concentrations and when it is produced by endogenous sources in cancer cells – can augment cancer progression by stimulating cancer cell growth, facilitating angiogenesis, and promoting resistance to chemotherapy [5, 6]. On the other hand, H_2_S – especially when applied exogenously, in the form of various H_2_S donor compounds – can also exert anti-tumor effects through induction of apoptosis and inhibition of cancer cell proliferation by reducing DNA synthesis and arresting the cell cycle [7, 8].

Several studies have demonstrated that overproduction of H_2_S occurs in breast cancer (BC) and correlated the upregulation of H_2_S synthesizing-enzymes, namely cystathionine-β-synthase (CBS) and cystathionine-γ-lyase (CSE), with poor clinical prognosis [9]. Wang et al., has shown that decreasing H_2_S level through CSE inhibition has led to inactivation of the JAK/STAT pathway via upregulation of SIRT1 [10]. You et al. have demonstrated a feedback regulation cycle between CSE and STAT3 and proposed a direct involvement of STAT3 in the amelioration of CSE transcription and H_2_S production [11]. CSE upregulation phosphorylates and activates STAT3 leading to augmentation of its own transcription in a positive-feedback manner [11]. CSE upregulation phosphorylates and activates STAT3 leading to augmentation of its own transcription in a positive-feedback manner.

One of the substantial physiological processes for H_2_S is the involvement in immunosurveillance. In recent years, extensive research has been carried out to determine the role of H_2_S in immunomodulation and in tumor immune microenvironment (TIME) [12]. Regulators of H_2_S-synthesizing machinery and H_2_S intracellular levels have been reported to modulate the TIME [13–15]. In a study conducted by our group, where BC cells were co-cultured with natural killer (NK) cells, the decrease of either CBS-derived or CSE-derived H_2_S has led to an increase in NK cell-mediated catalytic activity [15]. Moreover, CBS-derived H_2_S has exerted immunosuppressive activity by protecting BC cells from activated macrophage-generated ROS in macrophage-BC cell co-cultures [14]. As such, modulating H_2_S levels could also affect TIME. Dual inhibition of CBS and CSE in TNBC and HR + BC cells has affected the TIME by suppressing BC cells release of tumor necrosis factor alfa (TNF-α), a cytokine that acts as an immune-suppressor within the TIME [13]. Concomitantly, production of interferon gamma (IFN-γ) has restored the immune-stimulating conditions in the TIME [13].

3-Mercaptopyruvate sulfurtransferase (3MST), the third H_2_S-synthesizing enzyme, is well known for its critical physiological role in cellular metabolism and bioenergetics [16]. In recent years, there has been growing interest in the role of 3MST in cancer progression [17]. Some studies have shown that 3MST is upregulated in various types of cancers, including colon cancer [18, 19], glioma [20], lung carcinoma [21], renal cancer [22], oral cancer [23], as well as in glioblastoma cell lines [24, 25] but not adequately studied in BC.

The expression of various H_2_S biosynthetic enzymes can be directly controlled by miRNAs [26]. The expression profiles of miRNAs that target H_2_S-synthesizing enzymes, have been found to be altered in different clinical oncological and non-oncological settings. MiR-203 has been found to regulate oxidative stress induced cell injury by regulating CBS expression and adjusting the levels of H_2_S production [27]. In colorectal cancer, miR-559 was shown to target CBS thus reducing the accelerated cancer cell proliferation [28]. On the contrary, few miRNAs have exhibited dual or multiple targeting ability for H_2_S-synhesizing enzymes, like miR-4317 that targets CBS and CSE together [29]. Reports that describe miRNA targeting 3MST are not available. It was recently found by our research group that knocking down of CBS caused a compensatory increase in CSE expression rescuing H_2_S level [30]. This inhibition-sensory behavior highlights compensatory mechanisms that maintain the level of H_2_S in BC cells.

Given the key role of 3MST in fostering cancer cell survival and augmenting cancer cell growth and proliferation [2, 17, 25, 31], investigating its expression level in BC tissues could widen our understanding for the role of H_2_S synthesizing enzymes in BC tumorigenesis. Here, we screened in clinical breast cancer specimen the 3MST expression levels. Our data show that 3MST as well as CBS and CSE are significantly overexpressed in BC tissues compared to their non-cancerous counterparts. Furthermore, to escape the compensatory upregulation behavior of H_2_S-synthesizing enzymes, we performed *in-silico* analysis to achieve pan-inhibition of the three H_2_S synthases simultaneously. In a “three birds, one stone” approach, we show that utilizing one miRNA that targets CBS, CSE, 3MST simultaneously could markedly and efficiently suppress BC hallmarks in TNBC cells and enhance the expression of immunomodulatory factors.

## Materials and methods

### Clinical specimens

Breast tissues were collected from 25 BC female patients during conservative mastectomy or lumpectomy surgery at the National cancer Institute, Egypt. Tissues from both breast tumor and adjacent pathologically confirmed non-tumor tissues from the safety margins (5–7 cm away from the tumor margin) were resected. All specimens were confirmed in the Department of Pathology, and relevant clinical data were collected. BC patients who had a previous history of BC or smoking or hypertension were eliminated. The specimens were snap frozen in liquid nitrogen and immediately stored at -80 °C. The Ethics Committee of the Faculty of Pharmacy and Biotechnology, the German University in Cairo ratified the study protocol in accordance with the ethical standards of the Declaration of Helsinki. All individuals signed informed written consent documents prior to their involvement in the study.

### Bioinformatics analysis

*In-silico* bioinformatic analysis was carried out to identify novel miRNAs that have the potential to interact with the genes of interest; CBS, CSE, and 3MST as previously described in [32, 33]. Eight different bioinformatic websites have been used, namely, TargetScanHuman (www.targetscan.org/), miRDB (mirdb.org/), miRwalk (mirwalk.umm.uni-heidelberg.de/), miRIAD (www.miriad-database.org/), miRTar.human (ccb-web.cs.uni-saarland.de/mirtargetlink/), ComiRNet (comirnet.di.uniba.it:8080/interactionsSearch), FirePlex Discovery Engine (www.fireflybio.com/portal/search), and GeneCards (https://www.genecards.org/). Candidate miRNAs were selected based on miRNA-mRNA hybridization energy, complementary strength, binding score, seed match, and novelty in BC.

### Cell culture and treatment

Culture of human TNBC cell line MDA-MB-231 was conducted in Dulbecco’s modified Eagle’s medium (DMEM) (Lonza, Switzerland) supplemented with 4.5 g/l glucose, 4 mmol/l L glutamine, 10% fetal bovine serum (Lonza, Germany) and 1% Penicillin-Streptomycin (Lonza, Germany) at 37 °C in 5% CO_2_ atmosphere. Cells were passaged upon achieving 70–80% confluency. A stock solution of H_2_S donor (40 μm NaHS) was prepared using free DMEM. Co-treatment of the TNBC cells seeded in 96-well plates with the NaHS donor was performed for 24 h under normal growth conditions (37 °C in 5% CO_2_ atmosphere) [33, 34]. Control cells in the H_2_S donor experiments were subjected to DMEM only. All cell experiments in this study were performed in triplicate and repeated at least three times [26].

### Cell transfection

Different oligonucleotides including empty vector negative control scrambled miRNAs (Scr-miRNAs) (MSY0000449 - Qiagen, Germany), miR-193a-3p mimics (MSY0000459 - Qiagen, Germany), siRNA directed against human miR-193a-3p (Antagomir) (339,121, MIMAT0000459 - Qiagen, Germany), miR-548c-3p mimics (YM00473313-ADA - Qiagen, Germany), and siRNA directed against human miR-548c-3p (Antagomir) (339,121, MIMAT0003285 - Qiagen, Germany) were transfected into MDA-MB-231 cells. HiPerfect Transfection Reagent (Qiagen, Germany) was used in all transfection experiments [34–36].

### Reverse transcription-quantitative polymerase chain reaction (RT-qPCR)

Total RNA was extracted from BC patients and cell line using Biazol (Invitrogen, USA) reagent. The integrity of RNA was verified by gel electrophoresis on 1% agarose. For gene expression assay, RNA was reversely transcribed by the High-Capacity cDNA Reverse Transcription Kit (4,368,814 - ThermoFisher Scientific, USA) while for miRNA expression quantification, RNA was reversely transcribed by the TaqMan™ Advanced miRNA cDNA Synthesis Kit (A28007 - ThermoFisher Scientific, USA) according to the company’s instruction. RT-qPCR was performed in StepOne™ Plus (ABI, USA). All genes with their catalog number and assay ID are listed in Supplementary Table S1 in supplementary data. The housekeeping genes β-actin and 18s rRNA as well as miR-26b-5p were endogenous controls. The 2^−ΔΔCt^ method was applied to calculate relative expression [29, 37].

### Quantification of H_2_S production

H_2_S levels were measured using the H_2_S-sensitive fluorescent probe 7-azido-4-methylcoumarin (AzMC) (Sigma-Aldrich). TNBC cells were seeded in black 96-well plates with an optical bottom at 10,000 cells/well in 100 µl full DMEM and incubated at 37 °C and 5% CO_2_ to allow cells to adhere. After 24 h seeding, the cells were transfected with the oligonucleotides of interest. Then, 48 h post-transfection, the supernatant was replaced by 100 µl of 100 µM of AzMC (Sigma-Aldrich) prepared in HBSS. After one hour of incubation at 37 °C in the dark, fluorescence was measured on the Wallac 1420 Victor reader with excitation and emission wavelengths of 355 and 460 nm, respectively. The final concentration of DMSO was kept constant at 0.1% in all conditions. Data analysis was performed after removal of the non-specific background fluorescence values [38].

### Cellular viability assay (MTT assay)

Cellular viability was assessed using 3-(4,5-dimethylthiazol-2-yl)-2,5-diphenyltetrazolium bromide (MTT). After transfection and incubation of cells for 48 h in 96-well plates, supernatants were replaced with 100 µl of 0.05% of MTT working solution diluted in free DMEM and incubated for 4 h. Afterwards, cells were supplemented with 200 µL dimethyl sulfoxide (DMSO) and observed for dissolution of the formed formazan purple crystals. Absorbance was measured at 595 nm using the Wallac 1420 Victor reader [39].

### Colony forming assay

Transfected cells were counted by hemocytometer and fostered in 6-well plates at 0.4 × 10^3^ cells/well for 2 weeks. The formed cell colonies were fixed with 6% glutaraldehyde and stained with 0.2% crystal violet solution. Then, the stained cell colonies were manually counted [35].

### Scratch test (wound healing assay)

Transfected cells at 90–95% confluence in 24-well plates were scraped by a sterile 10 µl micropipette tip vertically along a ruler. Then, cells were rinsed with PBS to remove detached cells and substituted with new low serum media (1% FBS). Afterwards, cells were observed for the migration rate at 0 and 24 h and wound closure was quantified with Zen2012 software (ZEISS Microscopy, Jena, Germany) by measuring the surface area of the scratch [40, 41].

### Transwell migration assay (Boyden chamber assay)

Transfected cells were seeded into the upper part of the 5-µm Transwell chamber (cellQART®, Germany) at concentration of 5 × 10^4^ cells suspended in 300 µl of 1% full DMEM. The medium (700 µL) containing 10% FBS was added to the lower part. Through incubation under the conventional conditions, the non-migrating cells in the upper part were removed while the migrating cells in the lower part were fixed in 6% glutaraldehyde and stained with 0.2% crystal violet solution, followed by photography under an optical microscope. For accurate assessment, each insert was then transferred to an empty well containing 700 µl of the extraction solution (33% acetic acid) to lyse the cells, and 100 µl of each sample was transferred and measured at 595 nm using Wallac 1420 Victor reader.

### Statistical analysis

Sample size was calculated by G*Power version 3.1.9.2; Germany with a power 80% and a level of significance (α) of 5% and expected effect size (1-β) of 0.8. Data are presented in the form of mean ± standard error of the mean (SEM). Student’s t test was performed to compare between every two independent groups. Statistical significance was considered as *p* < 0.05. For multiple comparisons, one-way analysis of variance (One-way ANOVA) with post hoc analysis was used. Data were statistically analyzed using GraphPad Prism 8.00 software (GraphPad Software Inc., San Diego CA).

## Results

### CBS, CSE and 3MST are overexpressed in BC tissues

A summary of the patients’ characteristics is provided in Table 1. The average age of the BC patients was 46.36 years, with an age range of 26–72 years. According to molecular subtype, 52% of the patients were of luminal A subtype, 12% were luminal B, 28% were TNBC while only 8% were of HER2-enriched subtype. According to tumor grade, 8% of the patients had grade I BC, 64% had grade II while 28% had grade III. For lymphatic involvement, 60% of the patients had lymph node metastases. 80% of the patients expressed high proliferative index Ki-67 levels. A small number of participants (2/25; 8%) were identified to have the invasive/infiltrating lobular carcinoma (ILC) histological subtype. Additionally, 64%, 56%, and 16% of the patients showed positive expression of ER, PR, and HER2, respectively.

**Table 1:** Characteristic features of BC female patients

	BC patients	Percentage
**Age**	≥ 40	48%
	< 40	52%
**Grade**	I	8%
	II	64%
	III	28%
**Histological type**	Ductal	92%
	Lobular	28%
**Molecular subtype**	Luminal A	52%
	Luminal B	12%
	HER2 enriched	8%
	TNBC	28%
**ER status**	Positive	64%
	Negative	36%
**PR status**	Positive	56%
	Negative	44%
**HER-2 status**	Positive	16%
	Negative	84%
**Lymphatic involvement**	Yes	60%
	No	40%
**Proliferative index (Ki-67)**	High (> 14%)	80%
	Low (< 14%)	20%

**Fig. 1:**
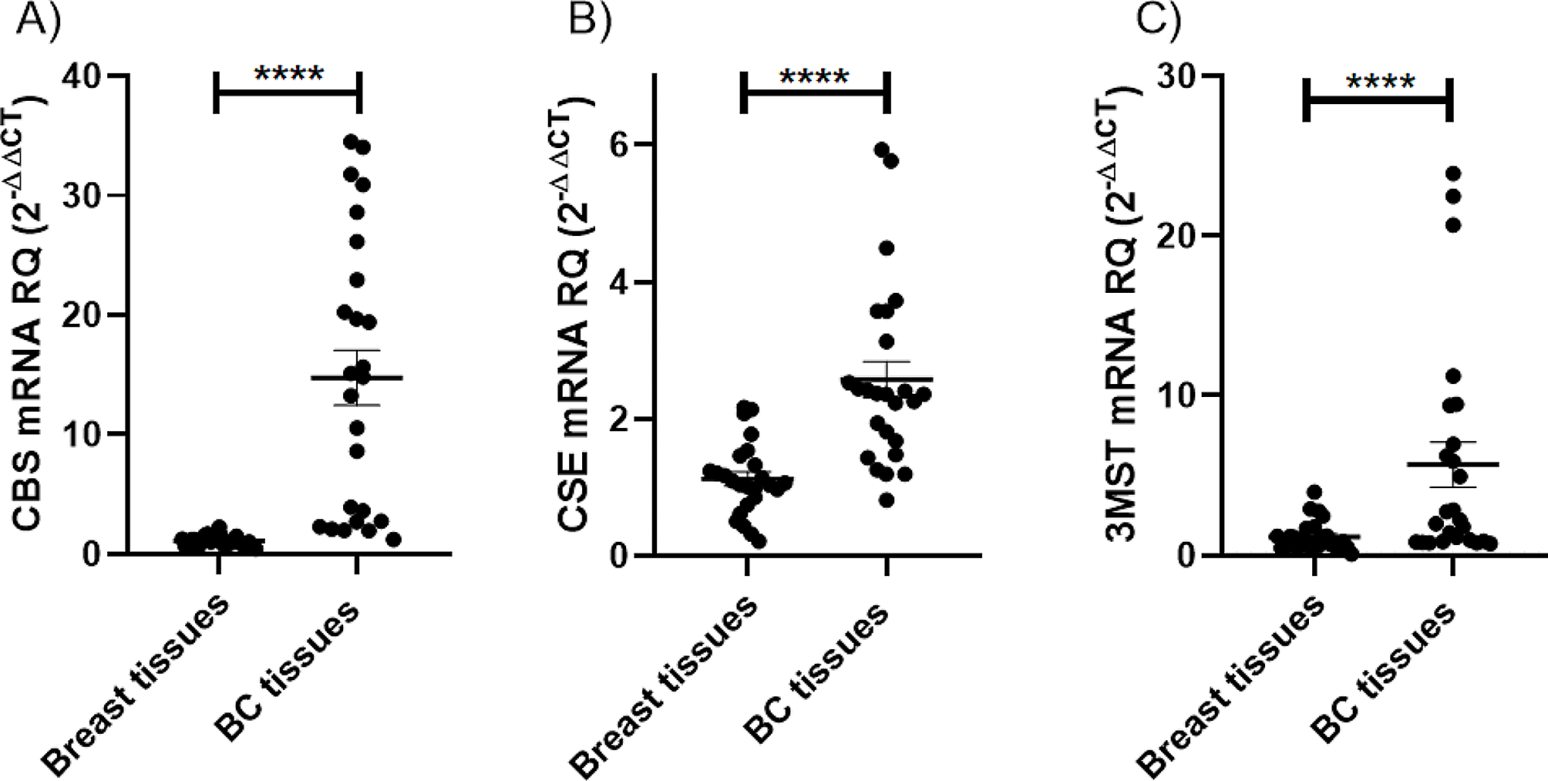
Screening of CBS, CSE, and 3MST in BC tissue. The expression profiles of the H_2_S-synthesizing enzymes, (**A**) CBS, (**B**) CSE, and (**C**) 3MST were analyzed in 25 BC patients using qRT-PCR and normalized to 18 S as an internal control. The three H_2_S-synthesizing enzymes showed a significant overexpression in BC tissues compared to their normal counterparts. Student t test was performed. **** = *P* < 0.0001 compared to normal counterparts

Screening of CBS, CSE, and 3MST expression levels in BC tissues displayed a marked overexpression in the transcript levels of CBS (*P* < 0.0001) (Fig. 1A), CSE (*P* < 0.0001) (Fig. 1B), and 3MST (*P* = 0.0038) (Fig. 1C), when compared to normal counterparts.

**Fig. 2:**
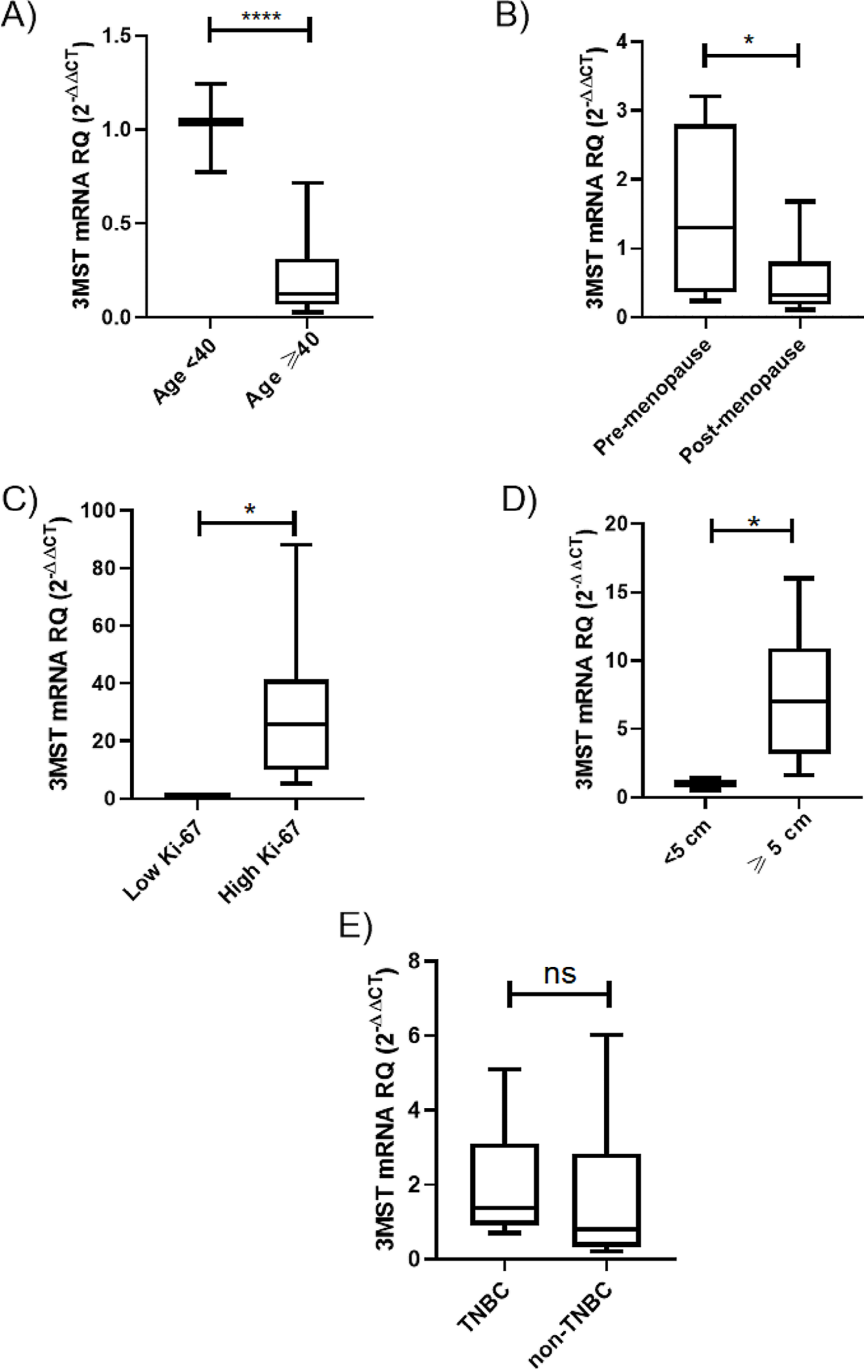
Stratification of 3MST expression in BC patients according to age, menopausal status, expression level of Ki-67, tumor size, and molecular subtype. Stratification of 3MST expression levels in BC patients based on (**A**) age, (**B**) menopausal status, (**C**) expression level of Ki-67, and (**D**) tumor size showed a correlation with the more aggressive profiles of BC. 3MST was found to be significantly overexpressed in BC patients < 40 years old, pre-menopause, who are expressing high Ki-67 levels and whose tumor size ≥ 5 cm. (**E**) 3MST levels were consistent among the different BC subtypes with no preference to TNBC. Student t test was performed. *= *P* < 0.05, **** = *P* < 0.0001

3MST expression data obtained from BC patients has been stratified according to different patient features such as age (< 40 or ≥ 40 years old), menopausal status (pre-menopause or post- menopause), expression level of Ki-67 (high or low), tumor size (< 5 or ≥ 5 cm) and molecular subtype. 3MST showed higher transcript level in BC patients at age younger than 40 years old (*P* < 0.0001) (Fig. 2A), in pre-menopausal status (*P* = 0.0469) (Fig. 2B), expressing high Ki-67 (*P* = 0.0401) (Fig. 2C) and having large tumor size (≥ 5 cm) (*P* = 0.0187) (Fig. 2D). 3MST expression pattern did not vary between TNBC and non-TNBC patients (Fig. 2E).

### In-silico analysis revealed miR-193a-3p and miR-548c-3p as the candidates of choice

The miRNA candidates selected to simultaneously target CBS, CSE, and 3MST were hsa-miR-193a-3p and has-miR-548c-3p. Their accession numbers and mature sequences were obtained (Supplementary Tables S2 and Table S4) and were introduced into the eight different computational algorithms. MiR-193a-3p was found to hit CBS 3’UTR sequence at 1 binding region, CSE CDS region at 2 different binding sites, and 3MST 3’UTR sequence at 1 binding region (Supplementary Table S3). MiR-548c-3p was found to hit CBS 3’UTR sequence at 6 different binding regions, CSE CDS at 9 different binding regions, and 3MST 3’UTR sequence at 1 binding region (Supplementary Table S5).

### miR-193a-3p and miR-548c-3p are underexpressed in BC tissues

**Fig. 3:**
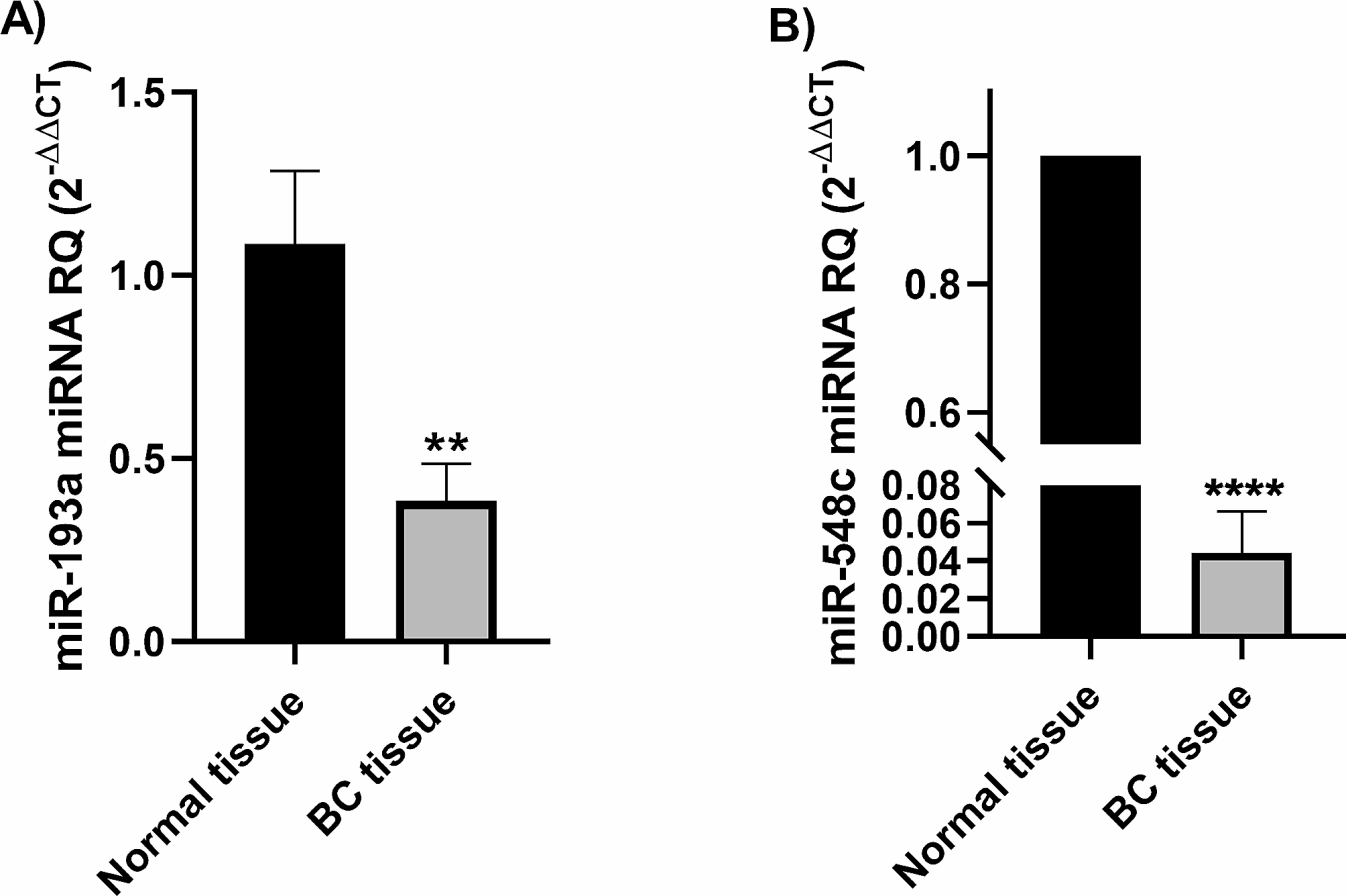
Expression profiles of miR-193a-3p and miR-548c-3p in BC patients. MiR-193a-3p and miR-548c-3p expression profiles were analyzed in 25 BC patients using qRT-PCR and normalized to miR-26b-5p as an internal control. Screening of miR-193a-3p (**A**) and miR-548c-3p (**B**) in breast tissues showed a significant under expression compared to normal counterparts.; **= *P* < 0.01, **** = *P* < 0.0001 compared to noncancerous breast tissues

Both miR-193a-3p and miR-548c-3p were significantly underexpressed in BC patients (*P* = 0.009) (Fig. 3A) and (*P* < 0.0001) (Fig. 3B), respectively.

### Ectopic expression of miR-193a-3p and miR-548c-3p in MDA-MB-231 cells suppresses CBS, CSE and 3MST transcription and reduces H_2_S levels

**Fig. 4:**
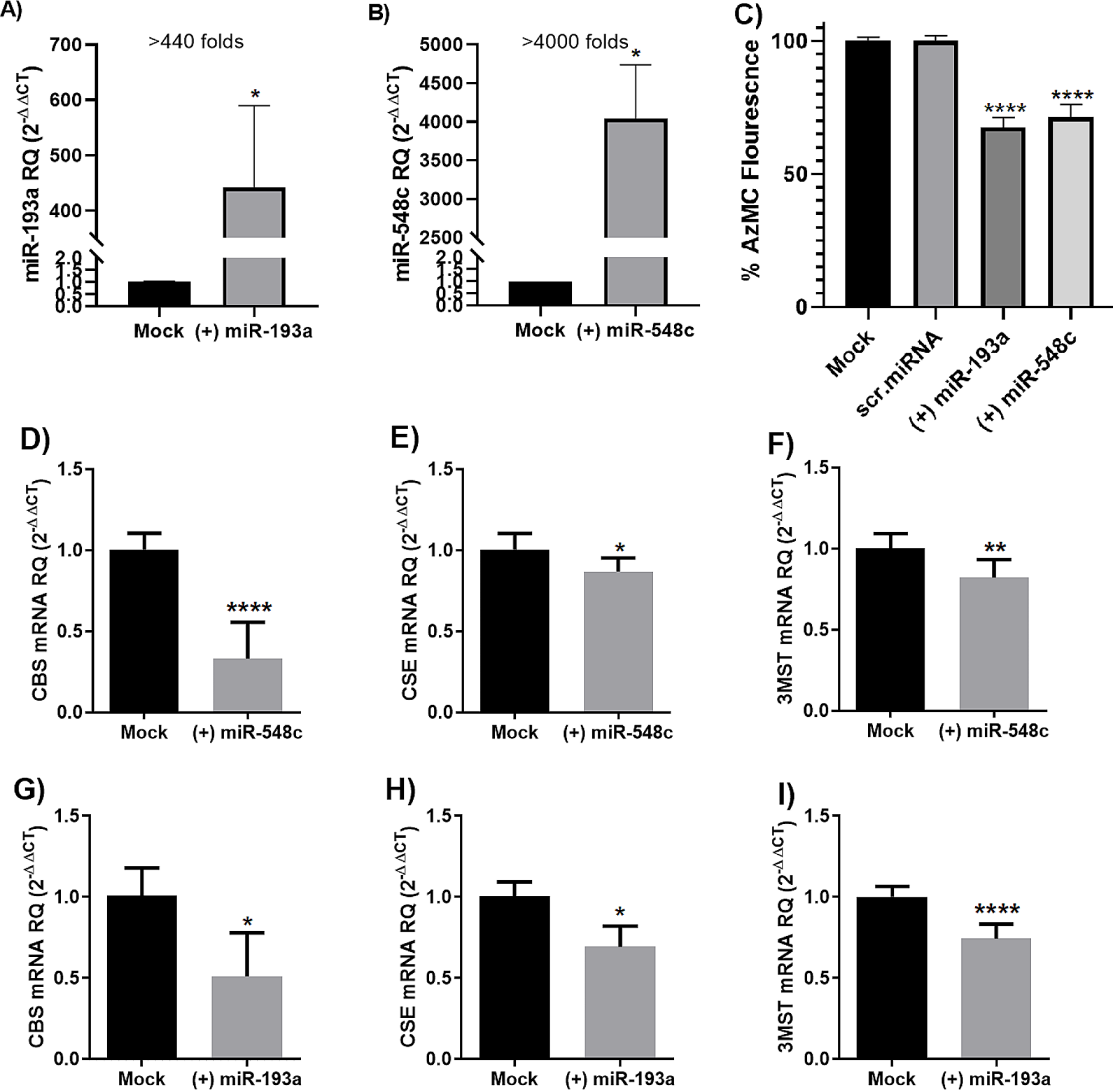
Impact of miR-193a-3p and miR-548c-3p transfection on CBS/CSE/3MST induced H_2_S production in MDA-MB-231 cells. (**A, B**) Efficient transfection of miR-193a-3p and miR-548c-3p oligonucleotides. (**C**) H_2_S levels. (**D, E,F**) CBS, CSE, and 3MST expression levels in miR-548c-3p transfected cells. (**G, H,I**) CBS, CSE and 3MST expression levels in miR-193a-3p transfected cells. *= *P* < 0.05, **= *P* < 0.01, **** = *P* < 0.0001 compared with control group

To evaluate the effect of miR-193a-3p and miR-548c-3p on H_2_S-synthesizing enzymes, MDA-MB-231 cells were transfected with miR-193a-3p and miR-548c-3p mimics. Transfection was validated by measuring the transcript levels of miR-193a-3p and miR-548c-3p 48 h post-transfection using qRT-PCR. Results showed a marked upregulation of miR-193a-3p and miR-548c-3p expression > 440 folds (*P* = 0.0404), and > 4000 folds (*P* = 0.0282), respectively, in MDA-MB-231 cells (Fig. 4A and B).

Ectopic expression of both miRNAs in MDA-MB-231 cells caused a significant suppression of H_2_S production (Fig. 4C) and reduction of CBS, CSE and 3MST transcript levels (Fig. 4D-I).

### Overexpression of miR-193a-3p and miR-548c-3p attenuates the oncogenic properties of TNBC cells

**Fig. 5:**
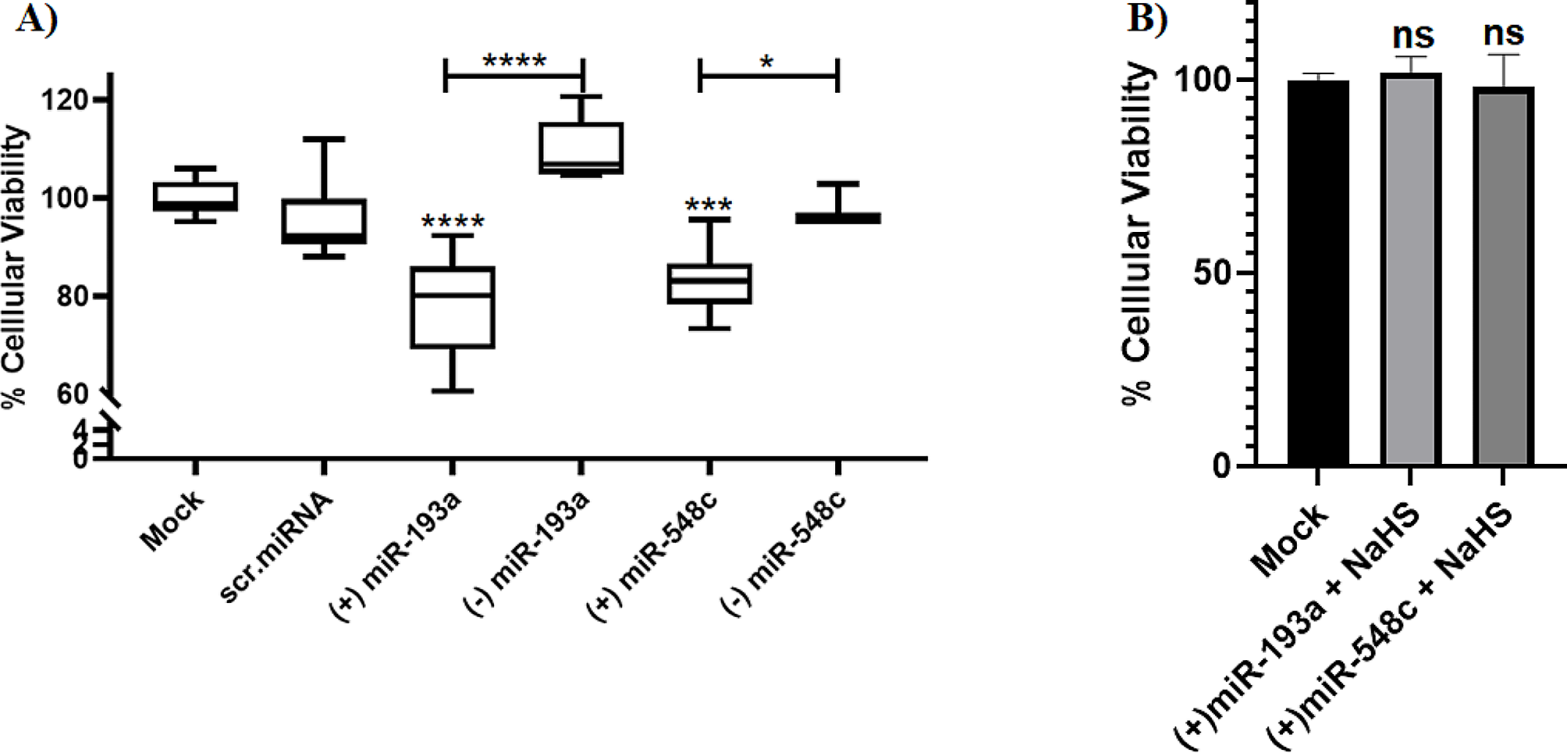
Impact of miR-193a-3p and miR-548c-3p overexpression on viability of MDA-MB-231 cells. (**A**) significant repression of cellular viability by miR-193a-3p and miR-548c-3p transfection as compared to mock and cells transfected with Scr-miRNAs. (**B**) Co-treatment of H_2_S donors with miR-193a-3p and miR-548c-3p mimic resulted in a total abrogation of miR-193a-3p and miR-548c-3p effects on cellular viability. One-way (ANOVA) multiple comparison was performed. *= *P* < 0.05, ***= *P* < 0.001, ****=*P* < 0.0001, ns = not significant compared with control groups

Forced expression of miR-193a-3p and miR-548c-3p in MDA-MB-231 cells resulted in a significant reduction in cellular viability of miR-193a-3p mimicked cells (*P* < 0.0001) and miR-548c-3p mimicked cells (*P* = 0.0005) (Fig. 5A). To validate that miR-193a-3p and miR-548c-3p induced suppression of cellular viability is due to repression of the endogenous H_2_S levels within TNBC cell line, MDA-MB-231 cells were transfected with miR-193a-3p or miR-548c-3p and co-treated with H_2_S donor (NaHS) at concentration of 10 µM NaHS/well. Total abrogation of miR-193a-3p and miR-548c-3p effects on cellular viability was observed (Fig. 5B).

**Fig. 6:**
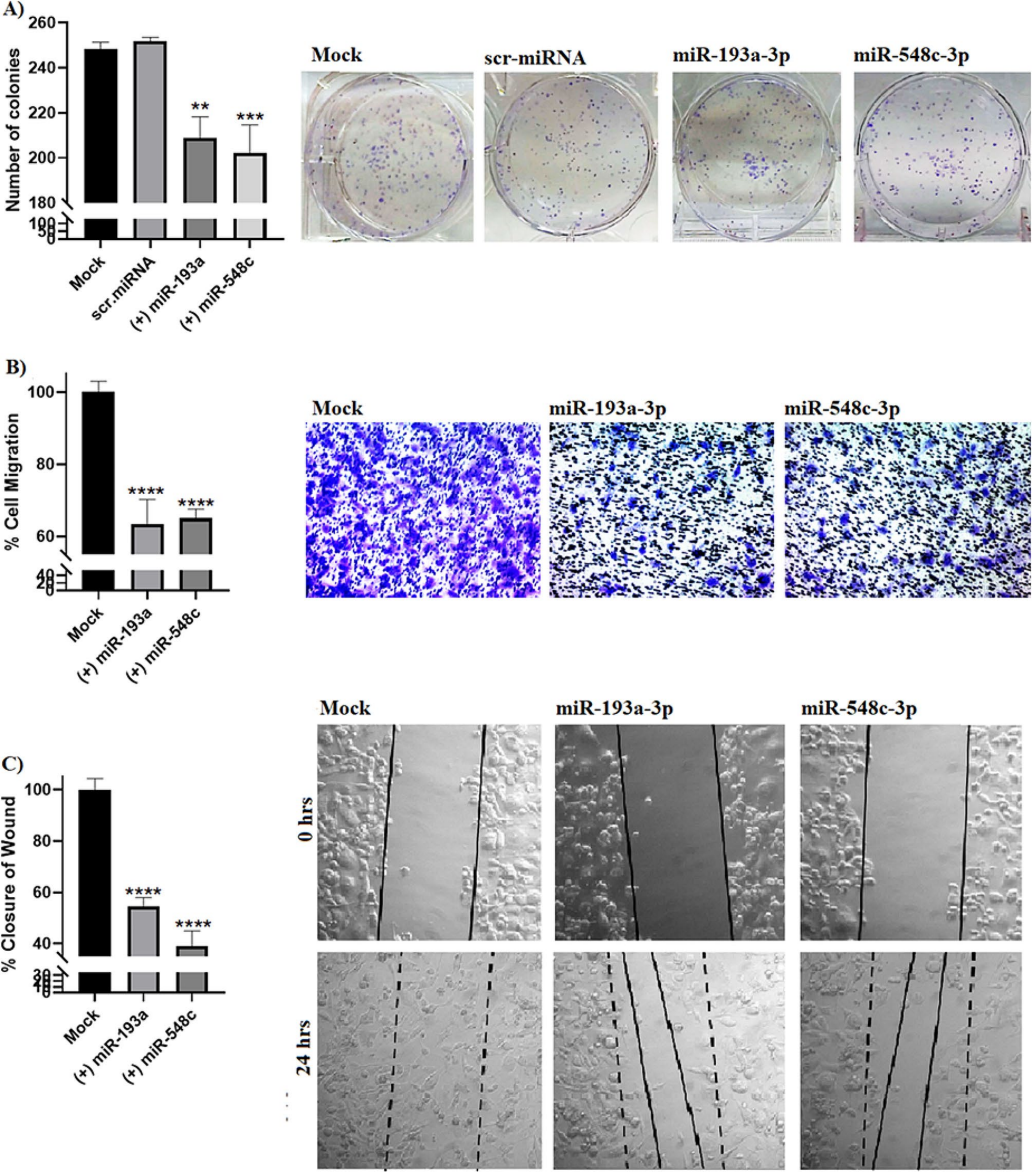
Impact of miR-193a-3p and miR-548c-3p overexpression on BC hallmarks in MDA-MB-231 cells. Ectopic expression of miR-193a-3p and miR-548c-3p in MDA-MB-231 cells resulted in a significant reduction in clonogenicity (**panel A**) migration ability (**panel B**) and wound healing (**panel C**). One-way analysis of variance (ANOVA) was performed. ****=*P* < 0.0001, ***= *P* < 0.001, **= *P* < 0.01 compared with control group

Regarding clonogenicity assay, the number and size of colonies decreased significantly for MDA-MB-231 cells transfected with miR-193a-3p (*P* = 0.0025) and miR-548c-3p (*P* = 0.0004) (Fig. 6A). In a similar pattern, ectopic expression of miR-193a-3p and miR-548c-3p in MDA-MB-231 cells caused a marked decrease in the cellular scratch healing capacity (*P* < 0.0001) (Fig. 6B) and migration ability (*P* < 0.0001) (Fig. 6C).

### Impact of miR-193a-3p and miR-548c-3p overexpression on immunogenic profile of TNBC cells

**Fig. 7:**
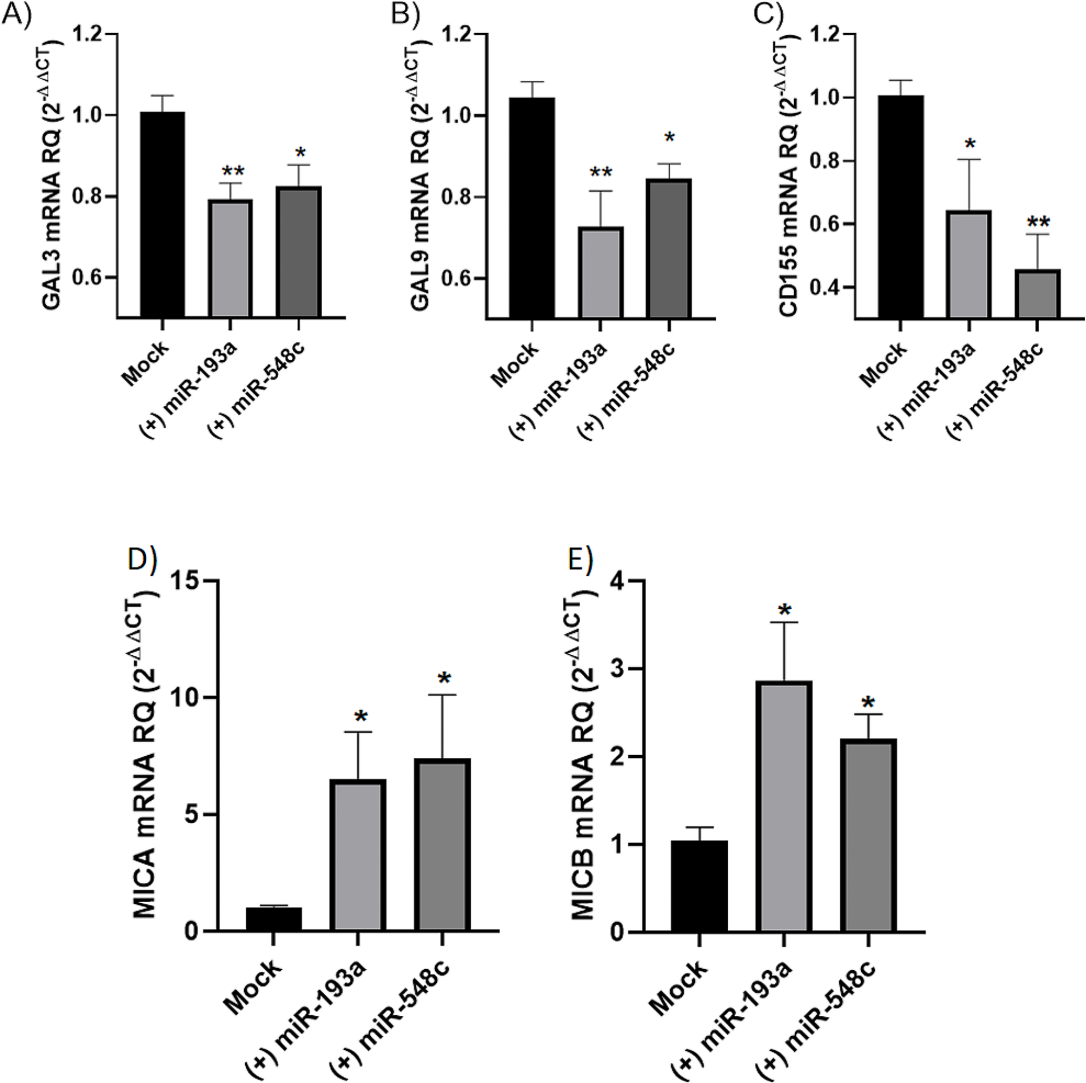
Impact of miR-193a-3p and miR-548c-3p transfection on immunogenic profile of TNBC cells. The expression levels of (**A**) GAL3, (**B**) GAL9, (**C**) CD155, (**D**) MICA, and (**E**) MICB in MDA-MB-231 cells transfected with miR-193a-3p and miR-548c-3p were determined 48 h post-transfection using qRT-PCR. Immunomodulatory factors expression levels were normalized to β actin as an internal control. One-way analysis of variance (ANOVA) was performed. *=*P* < 0.05, **= *P* < 0.01 compared with control group

Ectopic expression of miR-193a-3p and miR-548c-3p induced a marked decrease in the levels of the immunosuppressive GAL3 (*P* = 0.0089 and *P* = 0.0197) (Fig. 7A), GAL9 (*P* = 0.0013 and *P* = 0.0130) (Fig. 7B), and CD155 (*P* = 0.0469 and *P* = 0.0033) (Fig. 7C) transcript levels. In contrast, a marked upregulation of the immunostimulatory MICA (*P* = 0.0469 and *P* = 0.0285) (Fig. 7D) and MICB (*P* = 0.0154 and 0.0480) (Fig. 7E) was seen.

## Discussion

BC remains the most prevalent tumor among women worldwide among all age categories [42]. TNBC subtype of BC, which lacks ER, PR, and HER2 expression, is known as the most aggressive subtype of BC with a relatively high recurrence rate, especially within the first five years after diagnosis [43, 44]. It also has an increased tendency of metastasizing to other organs, including the liver, lungs, and brain [43]. The standard treatment for TNBC includes surgery, radiation therapy, and chemotherapy which is usually given before the surgery as a neoadjuvant [45]. However, TNBC is highly resistant to chemotherapy, making it more challenging to treat [46]. Significant efforts are being focused on the development of targeted and immunotherapies that may improve the prognosis of TNBC patients [46]. One such strategy involves using miRNAs [47]. Due to the promising results shown by several preclinical studies using miRNAs as therapeutic targets for TNBC [48], clinical trials are underway to evaluate the effectiveness of clinical translation of miRNA-based therapy into practice for treatment of different cancers, including TNBC [49].

H_2_S, the most recently identified gasotransmitter member, has been found to be involved not only in the regulation of various physiological processes, but also in many pathophysiological events such as tumor progression [17]. Dysregulation of H_2_S and its synthesizing enzymes have been linked to malignancies by either showing overexpression, or downregulation, highlighting that H_2_S and its synthesizing machinery have a tumor specific character [50, 51]. Our group has previously identified the upregulation of CBS and CSE on the transcriptional level either in cell line or in clinical samples of different BC subtypes [29]. These findings were supported by other studies that proved the involvement of H_2_S in many tumorigenic and immunosuppressive signaling pathways [52, 53]. Single or dual inhibition of CBS and CSE has significantly abrogated BC progression [29]. However, our group has recently observed [54] that, at single targeting of CBS, CSE gets upregulated as a compensatory mechanism to save the diminishment of H_2_S production. In the light of these findings, this study aimed to investigate the expression level of the third H_2_S-synthesizing enzyme, 3MST, along with the other two enzymes, in BC patients. Additionally, this work intended to find miRNAs that simultaneously target CBS, CSE, and 3MST to avert the compensatory upregulation response by the untargeted enzyme(s).

Screening of 3MST in BC tissues showed an overexpression of this enzyme in all BC subtypes. Overexpression pattern of 3MST was also reported in tumor tissues of brain gliomas [20], colon cancer [19], lung carcinoma [21], oral squamous cell carcinoma [23], adenoid cystic carcinoma of the oral cavity [55], renal cell carcinomas [22], and bladder urothelial cell carcinoma [56, 57], on the transcriptional level. This study was the first to demonstrate the association of 3MST with young age (< 40), pre-menopausal, large tumor size and high Ki BC patients. Lower 3MST expression levels were associated with larger tumor size in HCC [58]. CBS was also reported to be downregulated in HCC patients’ tissues [59]. One study by Kaczor-Kamińska and colleagues have screened 3MST in mouse mammary gland cell line (NMuMG) and mouse mammary gland tumor cell line (4T1) [60]. They found that relative gene expression of 3MST was higher in the tumor cell line when compared to the normal one.

After extensive *in silico* analysis, miR-193a-3p and miR-548c-3p were identified as miRNAs capable of triple targeting the three H_2_S-synthesizing enzymes. Upon testing the expression levels of the selected miRNAs in BC tissues, our findings revealed that they were prominently downregulated in all BC subtypes. Similar results for miR-193a-3p were observed in prostate cancer [61], bladder cancer [62], NSCLC [63], CRC [64], HER + BC [65], and ovarian cancer [66]. Paradoxically, other studies display miR-193a-3p as an oncomiR promoting radio resistance in nasopharyngeal cancer [67]. In agreement with our findings, miR-548c-3p showed tumor suppressor activity in bladder cancer [68], lung cancer [69], glioma [70], HCC [70], osteosarcoma [71, 72], BC [73], and papillary thyroid carcinoma [74]. On the contrary, other studies reported miR-548c-3p as an oncomiR in prostate cancer [75].

In support of the *in silico* work, we noticed that ectopic expression of miR-193a-3p and miR-548c-3p in TNBC cell line caused a reduction in the transcript levels of CBS, CSE and 3MST. Subsequently, a significant impediment in the H_2_S production level was observed. On a similar note, previous results demonstrated the impact of miR-193a-3p on repressing ERK protein, a validated downstream target of H_2_S [65]. Likewise, the impact of miR-548c-3p on HIF1-α, a signaling molecule for H_2_S, was reported by Du et al. [74]. Nonetheless, our study was the first to unravel the impact of miR-193a-3p and miR-548c-3p on impeding H_2_S production in TNBC through tribunal targeting of CBS, CSE, and 3MST.

**Fig. 8:**
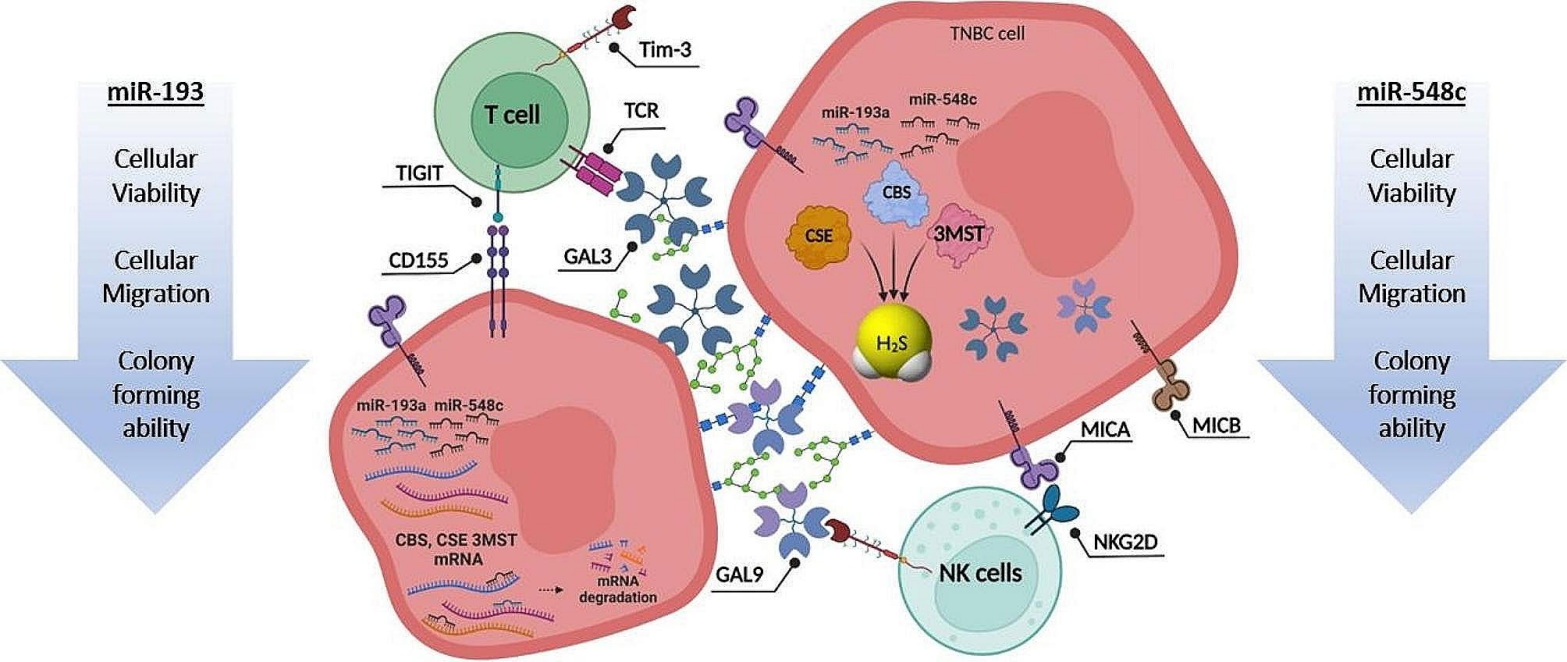
Pan-inhibition of the three H_2_S synthesizing enzymes by miRNA-193a and miRNA-548c modulates tumor cells and TME. Ectopic expression of miRNA-193a and miRNA-548c suppress the expression of CBS, CSE, and 3MST enzymes decreasing the H_2_S production level and accordingly TNBC hallmarks such as cellular viability, cellular migration and colony forming ability have been significantly repressed. MiRNA-193a and miRNA-548c also modulate the immune-modulators such as GAL3/9, MICA/B, and CD155

On the functional level, ectopic expression of miR-193a-3p and miR-548c-3p resulted in a marked reduction in multiple cancer hallmarks including cellular viability, migration ability, as well as clonogenicity of MDA-MB-231 cells as represented in Fig. 8. miR-193a-3p has been deemed as a tumor suppressor miRNA in NSCLC patients through inhibiting PAK4 via p53/Slug/L1CAM signaling pathways [63]. Likewise, miR-548c-3p has been reported as a tumor suppressor miRNA in osteosarcoma subjects through targeting ITGAV, alleviating cell proliferation [71]. In fact, ectopic overexpression of miR-548c-3p in osteosarcoma cell line has promoted apoptosis and G2/M cell cycle arrest, leading to abrogating the colony formation ability of the osteosarcoma cells [71]. This further advocates the role of miR-193a-3p and miR-548c-3p in BC progression, securing its position amongst other miRNAs which suppress BC progression such as let-7 [76], miR-4317 [77], miR-506-3p [39], and miR-486-5p [78].

Over the past decades, intensive efforts have been directed to find novel cancer therapies that attenuate the immunosuppressive and enhance the immunostimulatory effects against malignancy [79]. Indeed, immunomodulation is a promising approach in cancer therapy, including BC [80]. GAL3, GAL9, and CD155 are examples of molecules that play an immunosuppressant role, impeding the immune system’s ability to identify and attack cancer cells [81–84]. Thus, they represent potential targets for immunomodulatory therapies in cancer. GAL3 and GAL9 are molecules that are overproduced by cancer cells and can suppress the immune response by binding to TCR and Tim-3, respectively, which are expressed on T cell lymphocytes (TCLs) [84, 85]. Hence, inhibiting or blocking GAL3 and GAL9 can help to enhance the immune response against cancer cells. This conclusion is supported by the preclinical studies which have shown that targeting GAL3 or GAL9 could inhibit tumor growth and improve the efficacy of the immune checkpoint inhibitors (ICIs) [84, 86]. Moreover, CD155 is a protein that is overexpressed by some cancer cells preventing the activation of immune cells [87]. By blocking the CD155/TIGIT axis, the immune response can be enhanced and promote the elimination of cancer cells [88, 89]. Indeed, CD155 siRNAs can be effective in treating late-stage TNBC through immune escape [89]. Interestingly, a previous study from our group showed that modulation of H_2_S has a potential impact on the immunosurveillance process [13]. Our findings also have previously highlighted that dampening CBS and CSE expression levels using siRNAs consolidate the expression levels of MICA/B and ULBP2 in MDA-MB-231 cells, which in turn resulted in a marked increase in NK cells cytotoxicity upon co-culturing [90]. Herein, the overexpression of miR-193a-3p and miR-548c-3p reduced the expression level of GAL3, GAL9, and CD155 and upregulated the expression levels of MICA and MICB in MDA-MD-231. These results provide the first evidence of the involvement of miR-193a-3p and miR-548c-3p in improving NK cells and TCLs immunosurveillance via modulating H_2_S production in BC.

While our results offer promising insights, several limitations warrant consideration. Our study predominantly focused on the MDA-MB- 231 cell line, which may not fully capture the heterogeneity of BC in clinical populations. Further investigations encompassing diverse BC subtypes and clinical samples are needed to validate the translational potential of our findings. Additionally, elucidating the precise molecular mechanisms underpinning the pan-suppression of miR-193a-3p and miR-548c-3p on H_2_S synthesizing enzymes is essential.

## Conclusion

In conclusion, our study presents compelling evidence that the three H_2_S synthesizing enzymes CBS, CSE, and 3MST are overexpressed in BC. Upon patient stratification based on 3MST expression level, 3MST was found to have higher transcript levels in BC patients at age < 40 years old, pre-menopausal, expressing high Ki-67 and having large tumor size (≥ 5 cm). Bioinformatics and *in-silico* analysis predicted miR-193a-3p and miR-548c-3p as pan-suppressors of the three H_2_S synthesizing enzymes simultaneously. They were under-expressed in BC tissues. In-vitro study confirmed their pan-inhibition of the three H_2_S synthesizing in TNBC cells and attenuation of H_2_S production. Meanwhile, miR-193a-3p and miR-548c-3p suppressed the oncogenic profile of TNBCs and improved their immune response (Fig. 8).

### Electronic supplementary material

Below is the link to the electronic supplementary material.

Supplementary Material 1

## Data Availability

No datasets were generated or analysed during the current study.

## Abbreviations

H_2_S: Hydrogen sulfide
CBS: Cystathionine-β-synthase
CSE: Cystathionine-γ-lyase
3MST: 3-mercaptopyruvate sulfurtransferase
BC: Breast cancer
TNBC: Triple negative breast cancer
ER: Estrogen receptors
PR: Progesterone receptors
HER2: Epidermal growth factor
miRNA-193a: microRNA-193a-3p
miRNA-548c: microRNA-548c-3p
TIME: Tumor immune microenvironment
NK: Natural killer
TNF-α: Tumor necrosis factor alfa
IFN-γ: Interferon gamma
AzMC: 7-azido-4-methylcoumarin
ILC: Invasive/infiltrating lobular carcinoma
MICA/B: MHC class I chain-related protein A/B
ULBP2: UL16 binding protein 2
GAL 3/9: Galectin-3/9
