# Supplementary material for: Pan-inhibition of the three H2S synthesizing enzymes restrains tumor progression and immunosuppression in breast cancer
Source: Cancer Cell Int. 2024 Apr 16;24:136. doi: 10.1186/s12935-024-03317-1 (PMC11020979; doi:10.1186/s12935-024-03317-1)
Supplement: Supplementary file 1 — Supplementary Material 1 [file 12935_2024_3317_MOESM1_ESM.docx]

# **Supplementary data**

**Supplementary Table S1: Quantified genes with their catalog number and assay ID**

| Gene of interest | Catalog number | assay ID |
| --- | --- | --- |
| β-actin | 4448484 | Hs01060665_g1 |
| 18s rRNA | 4448484 | Hs99999901_s1 |
| CBS | 4331182 | Hs00163925_m1 |
| CSE | 4331182 | Hs00542284_m1 |
| 3MST | 4331182 | Hs05579360_s1 |
| GAL3 | 4331182 | Hs00173587_m1 |
| GAL9 | 4351372 | Hs01088493_g1 |
| CD155 | 4331182 | Hs00197846_m1 |
| MICA | 4331182 | Hs00741286_m1 |
| MICB | 4331182 | Hs00792952_m1 |
| miR-26b-5p | A25576 | rno481461_mir |
| miR-193a-3p | A25576 | 478306_mir |
| miR-548c-3p | A25576 | 479537_mir |

**Supplementary Table S2: Accession number and mature sequence of miR-193a-3p**

|  | hsa-miR-193a-3p |
| --- | --- |
| Accession number | MIMAT0000459 |
| Mature sequence. | 5’- AACUGGCCUACAAAGUCCCAGU-3’ |

**Supplementary Table S3: Binding regions on the 3’UTR or CDS of the target transcript CBS, CSE (CTH), and 3MST (MPST) aligned with the seed sequence of miR-193a-3p**

|  | Predicted consequential pairing of target region (top) and miRNA (bottom) | Site type |
| --- | --- | --- |
| Position 2070-2075 of CBS 3' UTR  hsa-miR-193a-3p | 5'.. UAUUGAGAGAGAAGUCGGCCAGG...  \|\|\|\|\|\|  3' ...UGACCCUGAAACAUCCGGUCAA | 6mer |
| Position 220-225 of CTH CDS  hsa-miR-193a-3p | 5'.. GCAGGCGAUCCAUGUGGGCCAGG...  \|\|\|\|\|\|  3' ...UGACCCUGAAACAUCCGGUCAA | 6mer |
| Position 301-306 of CTH CDS  hsa-miR-193a-3p | 5'.. CAAGCAAGGGGCGCCUGGCCAGG...  \|\|\|\|\|\|  3' ...UGACCCUGAAACAUCCGGUCAA | 6mer |
| Position 1054-1060 of MPST 3' UTR  hsa-miR-193a-3p | 5'... GGGGUGACAUCUCAAAGGCCAGG..  \|\|\|\|\|\|\|  3' ...UGACCCUGAAACAUCCGGUCAA | 7mer-m8 |

**Supplementary Table S4: Accession number and mature sequence of miR-548c-3p**

|  | hsa-miR-548c-3p |
| --- | --- |
| Accession number | MIMAT0003285 |
| Mature sequence. | 5’- CAAAAAUCUCAAUUACUUUUGC-3’ |

**Supplementary Table S5: Binding regions on the 3’UTR of the target transcript CBS, CSE (CTH), and 3MST (MPST) aligned with the seed sequence of miR-548c-3p**

|  | Predicted consequential pairing of target region (top) and miRNA (bottom) | Site type |
| --- | --- | --- |
| Position 2015-2021 of CBS 3' UTR  hsa-miR-548c-3p | 5'..CUGGCUAAUUUUUGUAUUUUUAC...  \|\|\|\|\| \|\|\|\|\|\|  3'CGUUUUCAUUAACUC--UAAAAAC | 7mer-A1 |
| Position 2150-2156 of CBS 3' UTR  hsa-miR-548c-3p | 5'..CGUCUACUUUUUAAUAUUUUUAG...  \|\|\|\|\|\|  3' CGUUUUCAUUAACUCUAAAAAC | 7mer-A1 |
| Position 2278-2284 of CBS 3' UTR  hsa-miR-548c-3p | 5'..ACUGCGCCUCGUCUAAUUUUUAA...  \|\|\|\|\|\|  3' CGUUUUCAUUAACUCUAAAAAC | 7mer-A1 |
| Position 2287-2293 of CBS 3' UTR  hsa-miR-548c-3p | 5'..CGUCUAAUUUUUAAUAUUUUUAG...  \|\|\|\|\| \|\|\|\|\|\|  3'GUUUUCAUUAACUC---UAAAAAC | 7mer-A1 |
| Position 2415-2421 of CBS 3' UTR  hsa-miR-548c-3p | 5'..ACUGCGCCUCGUCUAAUUUUUAA...  \|\|\|\|\|\|  3' CGUUUUCAUUAACUCUAAAAAC | 7mer-A1 |
| Position 2424-2430 of CBS 3' UTR  hsa-miR-548c-3p | 5'..CGUCUAAUUUUUAAUAUUUUUAG...  \|\|\|\|\| \|\|\|\|\|\|  3'GUUUUCAUUAACUC---UAAAAAC | 7mer-A1 |
| Position 392-398 of CTH 3' UTR  hsa-miR-548c-3p | 5'..UCUUAAAUCAAGUGUGAUUUUUU...  \|\|\|\|\|\|\|  3' CGUUUUCAUUAACUCUAAAAAC | 7mer-m8 |
| Position 517-523 of CTH 3' UTR  hsa-miR-548c-3p | 5'..AAGACUAUACUUAAUAUUUUUAA...  \|\|\|\|\|\|  3' CGUUUUCAUUAACUCUAAAAAC | 7mer-A1 |
| Position 950-956 of CTH 3' UTR  hsa-miR-548c-3p | 5'..UAGCCUUUUAUGUUAAUUUUUAU...  \|\|\|\|\|\|  3' CGUUUUCAUUAACUCUAAAAAC | 7mer-A1 |
| Position 1070-1076 of CTH 3' UTR  hsa-miR-548c-3p | 5'..AAUAGUACUUUUUAUAUUUUUAU...  \|\|\|\| \|\|\|\|\|\|  3'CGUUUUCAUUAACUC--UAAAAAC | 7mer-A1 |
| Position 3829-3835 of CTH 3' UTR  hsa-miR-548c-3p | 5'..AAAAAUUAAGACAUCAUUUUUAA...  \|\|\| \|\|\|\|\|\|  3' CGUUUUCAUUAACUC-UAAAAAC | 7mer-A1 |
| Position 4003-4009 of CTH 3' UTR  hsa-miR-548c-3p | 5'..CAAAUCUUAAUAGU-AUUUUUAU...  \|\|\|\| \|\|\|\|\|\|  3' CGUUUUCAUUAACUCUAAAAAC | 7mer-A1 |
| Position 4588-4594 of CTH 3' UTR  hsa-miR-548c-3p | 5'..UCUGACUUCAUUACUAUUUUUAC...  \|\|\|\|\|\|  3' CGUUUUCAUUAACUCUAAAAAC | 7mer-A1 |
| Position 4643-4649 of CTH 3' UTR  hsa-miR-548c-3p | 5'..AAAAAAAUCGGGCCUGAUUUUUU...  \|\|\|\|\|\|\|  3' CGUUUUCAUUAACUCUAAAAAC | 7mer-m8 |
| Position 5957-5963 of CTH 3' UTR  hsa-miR-548c-3p | 5'..CCGGCUAACUUUUAUAUUUUUAG...  \|\|\| \|\|\|\|\|\|  3'CGUUUUCAUUAACUC--UAAAAAC | 7mer-A1 |
| Position 1190-1195 of MPST 3'UTR  hsa-miR-548c-3p | 5’..CCUCCUUUCUGUUUUAUUUUUGA..  \|\|\|\|\|\|\|  3' CGUUUUCAUUAACUCUAAAAAC | 7mer-A1 |
